# Immunoprecipitation and mass spectrometry identify non-cell autonomous Otx2 homeoprotein in the granular and supragranular layers of mouse visual cortex

**DOI:** 10.12688/f1000research.4869.1

**Published:** 2014-07-30

**Authors:** Namsuk Kim, Dario Acampora, Florent Dingli, Damarys Loew, Antonio Simeone, Alain Prochiantz, Ariel A. Di Nardo

**Affiliations:** 1 CIRB, CNRS UMR 7241 / INSERM U1050, College de France, 11 place Marcelin Berthelot, 75231 Paris Cedex 05, France; 2 Institute of Genetics and Biophysics, Via Pietro Castellino 111, 80131 Napoli, Italy; 3 IRCCS Neuromed, 86077 Pozzilli (IS), Italy; 4 Institut Curie, Centre de Recherche, Laboratoire de Spectrométrie de Masse Protéomique, 75248 Paris Cedex 05, France

## Abstract

Plasticity in the visual cerebral cortex is regulated by the internalization of Otx2 homeoprotein into parvalbumin neurons in cortical layers II/III and IV. However the *Otx2* locus is not active in these neurons and the protein is imported from external sources, including the choroid plexus. Because Otx1 and Otx2 may have redundant functions, we wanted to verify if part of the staining in parvalbumin neurons corresponds to Otx1 transported from cortical layer V neurons. It is demonstrated here that Otx staining in layer IV cells is maintained in *Otx1*-null mice. The immunoprecipitation of extracts from finely dissected granular and supragranular cortex (layers I-IV) gave immunoblots with a band corresponding to Otx2 and not Otx1. Moreover, high-resolution mass spectrometry analysis after immunoprecipitation identifies two peptides within the Otx2 homeodomain. One of these peptides is specific for Otx2 and is not found in Otx1. These results unambiguously establish that the staining in parvalbumin neurons revealed with the anti-Otx2 antibodies used in our previous studies identifies non-cell autonomous Otx2.

## Introduction

Neural circuits generated during embryonic development are remodeled by environmental inputs during periods of heightened plasticity in postnatal development ^1^. These critical periods are limited to specific windows of time that are different for each sensory system. In the visual system, the primary visual cortex is subjected to a critical period for ocular dominance plasticity during which connections from a weak or absent eye can be permanently overtaken by the strong eye ^2^. The integrated action between inhibitory and excitatory circuits determines critical period onset, with a major role being played by the maturation of fast-spiking parvalbumin (FSPV) interneurons ^3^. We have shown that Otx2 homeoprotein helps determine critical period timing by signaling FSPV cells in mice ^4^. Conditional knock-down in heterozygous floxed mice just prior to normal critical period timing is sufficient to delay onset, while cortical infusion of recombinant Otx2 protein accelerates both onset and closure ^4^.

Remarkably, Otx2 protein in the cortex is non-cell autonomous. The *Otx2* locus is silent, as shown by PCR, *in situ* hybridization and *Otx2 ^+/GFP^* mice, while Otx2 protein is detectable by immunohistochemistry and immunoblot ^4, 5^. We have shown that cortical infusion of recombinant Otx2 protein results in specific uptake by FSPV cells, while injection in the retina results in its transport along the visual pathway and into these same cells ^4^. Blocking extracellular Otx2 through infusion of antibodies or specific peptides reduces uptake of endogenous Otx2 in FSPV cells ^4, 6^. Furthermore, we recently showed that the choroid plexus expresses Otx2 and secretes it into the cerebrospinal fluid ^5^. Conditional knockdown of *Otx2* expression in the choroid plexus results in reduced cortical levels of Otx2 protein. Needless to say, all of these approaches that alter Otx2 protein levels in the visual cortex have resulted in changes in cortical plasticity timing ^7^.

An outstanding question is whether cortical Otx1 homeoprotein also plays a role in the critical period. Indeed, Otx1 is expressed in the cerebral cortex during development and continues to be expressed by layer V neurons in the adult ^8^. It is thus possible that it is secreted by layer V cells and transferred into above granular and supragranular layers where Otx2 protein is detected. Unfortunately, most antibodies (commercial and academic) for Otx1 and Otx2 are pan-Otx thereby ruling out immunohistochemical approaches for conclusive evidence. Since genetic manipulation of the *Otx2* locus has resulted in reduced protein in layer IV visual cortex ^7^, we sought confirmation whether Otx1 is also present in these layers in the adult by using *Otx1* knockout mice and proteomic approaches.

## Methods

### Animals

All experiments were conducted in accordance with European Union Council Directives (86/609/EEC) and conform to Directive 2010/63/EU of the European Parliament. This study falls under project #00704.02 authorized by the French Ministry of Research. Adult male C57Bl/6J mice (Janvier) aged 2 to 3 months were used throughout this study and euthanized by cervical dislocation. *Otx1* knockout mice, in which *Otx1* is replaced with *lacZ* gene, have been previously described ^14^. Adult (~3 months) male *Otx1 ^LacZ/LacZ^* mice were fixed by intracardiac perfusion.

### Quantitative RT-PCR

Total RNA from frozen tissue samples were extracted by using RNeasy Mini kits (Qiagen) and were reverse transcribed (~400 ng) with Superscript II and oligo-dT primers (Invitrogen). For real-time PCR, samples were analyzed with a LightCycler Instrument (Roche) and SYBR green (Sigma) detection with the following primers:

Otx2-forward ATTCACGTTTCATGACCAACAG;

Otx2-reverse ATTGACTCCGTATGAGCGGTAT;

Otx1-forward GAACCTTCCTTCTCCGAAATCT;

Otx1-reverse GATCTTCACATCGGACAAATCA;

GAPDH-forward TGACGTGCCGCCTGGAGAAAC;

GAPDH-reverse CCGGCATCGAAGGTGGAAGAG.

The experiment was performed in duplicate. For calculating fold expression, gene-to-GAPDH ratios were determined by using the 2 ^-∆∆Ct^ method referenced to Otx1 expression in the lateral geniculate nucleus (LGN).

### Immunoprecipitation and immunoblots

Dissected tissue was lysed in 100 mM Tris-HCl, pH 7.4, 10 mM EDTA, 150 mM NaCl, 1% Triton X-100, and protease inhibitor (Roche) then triturated (22G and 26G needles). Lysates were centrifuged at 16,000 × g for 10 min at 4°C and supernatants were incubated either directly with antibodies or with antibody-coupled Dynabeads (Life Technologies) at 4°C for 16 h. For samples with uncoupled antibodies, Protein A Dynabeads (Life Technologies) were added and incubated at 4°C for 1 h. Proteins were eluted with 2X SDS sample buffer after five washes with lysis buffer. Antibodies were rabbit polycolonal IgG (Abcam ab27478) and anti-Otx2 (rabbit polyclonal, Abcam ab21990) and were coupled to Dynabeads according to manufacturer instructions (Life Technologies). For immunoblots, samples were separated on NuPAGE 4%–12% Bis-Tris precast gels (Invitrogen) and transferred onto PVDF membrane. Membranes were incubated overnight at 4°C with anti-Otx2 (rabbit polyclonal, 1/1,000, Abcam ab21990) and then with HRP-coupled anti-rabbit IgG (1/2,000, GE Healthcare NA934) 1 h at RT.

### Immunohistochemistry

For immunostaining, 50 µm floating sections were incubated with anti-Otx2 (rat polyclonal, 1/200, in-house) and biotinylated-WFA (1/100, Sigma L1516) in TBS, 1% Triton-X, 0.2% Tween-20, 10% fetal calf serum, overnight at 4°C. Sections were extensively washed at RT, incubated 2 h at RT with anti-rat Alexa Fluor-488 (Molecular Probes A21208, 1/2,000) and streptavidin Alexa Fluor-546 (Molecular Probes S11225, 1/2,000), washed again and finally mounted in Fluoromount medium (SouthernBiotech). Images were acquired with an Eclipse 90i microscope (Nikon).

### Mass spectrometry analysis

After immunoprecipitation, proteins were separated on SDS–PAGE gels (Invitrogen) and stained with colloidal blue staining (LabSafe GEL BlueTM GBiosciences). Gel slices were excised and proteins were reduced with 10 mM DTT prior to alkylation with 55 mM iodoacetamide. After washing and shrinking the gel pieces with 100% MeCN, in-gel digestion was performed using trypsin (Promega) overnight in 25 mM NH4HCO3 at 30°C.

Peptides were extracted and analyzed by nano-LC-MS/MS using an Ultimate 3000 system (Dionex S.A.) coupled to an Orbitrap Fusion mass spectrometer (Q-OT-qIT, Thermo Fisher Scientific). Samples were loaded on a C18 precolumn (300 µm inner diameter × 5 mm; Dionex) at 20 µl/min in 5% MeCN, 0.1% TFA. After a desalting for 3 min, the precolumn was switched on the C18 column (75 μm i.d. × 50 cm, packed with C18 PepMap™, 3 μm, 100 Å; LC Packings) equilibrated in solvent A (2% MeCN, 0.1% HCO2H). Bound peptides were eluted using a 150 min linear gradient (from 5 to 30% (v/v)) of solvent B (80% MeCN, 0.085% HCO2H) at a 150 nl/min flow rate and an oven temperature of 40°C. We acquired Survey MS scans in the Orbitrap on the 400–1500 m/z range with the resolution set to a value of 240,000 and a 4 × 105 ion count target. Each scan was recalibrated in real time by co-injecting an internal standard from ambient air into the C-trap. Tandem MS was performed by isolation at 1.6 Th with the quadrupole, HCD fragmentation with normalized collision energy of 35, and rapid scan MS analysis in the ion trap. The MS2 ion count target was set to 104 and the max injection time was 200 ms. Only those precursors with charge state 2–7 were sampled for MS2. The dynamic exclusion duration was set to 60 s with a 10 ppm tolerance around the selected precursor and its isotopes. The instrument was run in top speed mode with 3 s cycles.

Data were acquired using the Xcalibur software (v 3.0.63) and the resulting spectra were interrogated by the MascotTM Software through Proteome Discoverer (v 1.4.0.288, Thermo Scientific) with the SwissProt Mus musculus database (20140402, 16,671 sequences). We set carbamidomethyle cysteine, oxidation of methionine and N-terminal acetylation as variable modifications. We set specificity of trypsin digestion and allowed 2 missed cleavage sites and we set the mass tolerances in MS and MS/MS to 2 ppm and 0.5 Da, respectively. The resulting Mascot files were further processed by using myProMS (v 3.0) ^15^ and the estimated false discovery rate (FDR) by automatically filtering the Mascot score of all peptide identifications was less than 0.5%.

## Results

Quantitative PCR and mass spectrometry data of Otx2 homeoprotein in the mouse visual cortex **qPCR data**: This excel file (“brain qpcr otx data”) contains two worksheets. One worksheet has the Ct values for GAPDH, Otx2 and Otx1 obtained from extracts of different brain structures (LGN, Visual Cortex, Superior Colliculus and Cerebellum). Also included are the delta-Ct calculations. The second sheet contains the normalisation calculations and corresponding graph of expression values.
**Mass spectroscoy data**: This Mascot .dat file (“F022130_part.dat”) contains the experiment description and the data pertaining to the 2 peptides that match Otx2 protein.

### Otx1 locus but not Otx2 locus is active in the mouse visual cortex

**Figure 1.**
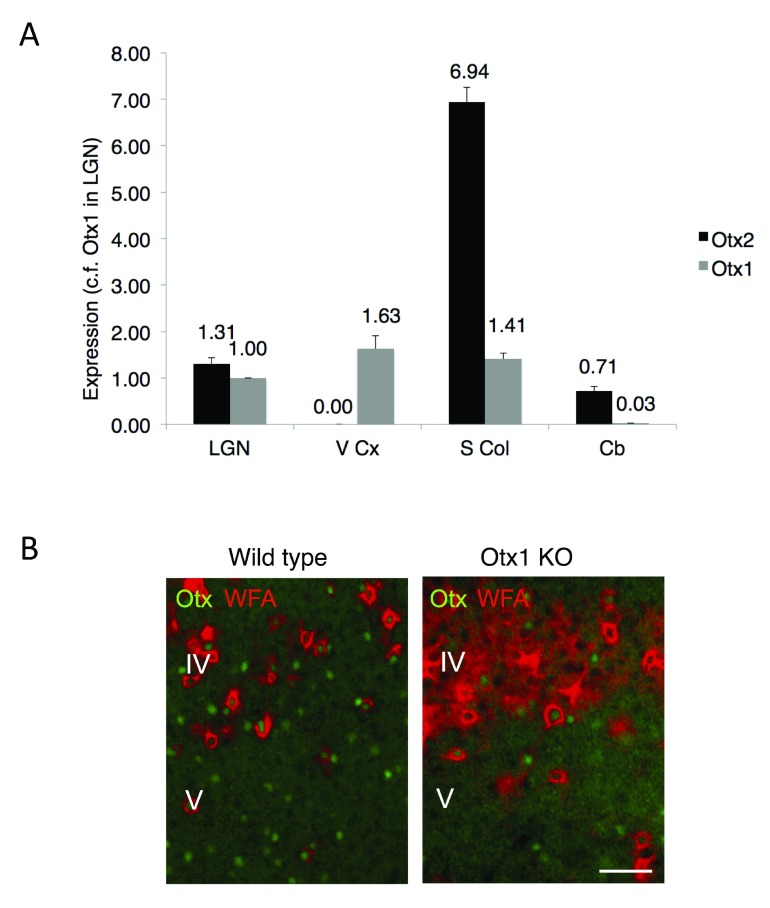
Expression of Otx1 and Otx2 in adult mouse brain. ( **A**) Analysis of Otx1 and Otx2 expression by quantitative RT-PCR on extracts from lateral geniculate nucleus (LGN), visual cortex (V Cx), superior colliculus (S Col) and cerebellum (Cb). The fold-difference in expression is calculated relative to Otx1 in LGN. The *Otx2* locus is silent in visual cortex. ( **B**) Non-cell autonomous Otx2 is found in visual cortex. Immunostaining in wild type mice reveals Otx1/2 cells in layers IV and V of visual cortex, including cells with perineuronal nets (stained by WFA lectin) enriched in layer IV. Staining for Otx2 persists in *Otx1* null mice (Otx1 KO). Scale bar, 50 µm.

We compared the expression of the *Otx1* and *Otx2* loci of various brain regions by performing quantitative PCR ( Figure 1A). While both mRNA were detected in thalamic and cerebellar structures, only Otx1 mRNA was found in the visual cortex. This result confirms the previously reported absence of GFP expression in the visual cortex of *Otx2 ^+/GFP^* knockout mice and lack of signal in the visual cortex of wild type mice after Otx2 *in situ* hybridization, even though Otx2 antibodies label FSPV cells ^4^. These cells are enwrapped by a dense extracellular matrix called perineuronal nets (PNNs) when localized to layer IV of the cortex ( Figure 1B). *Otx1* locus is active in layer V ^8^. In immunohistochemical analysis of *Otx1* knockout mouse, almost all Otx signal is lost in layer V while signal from Otx2 protein continues in PNN-labeled cells ( Figure 1B). However, we cannot preclude that some Otx1 is also present in layer IV cells in wild type mice.

### Otx2 protein but not Otx1 protein is found in granular and supragranular layers of visual cortex

**Figure 2.**
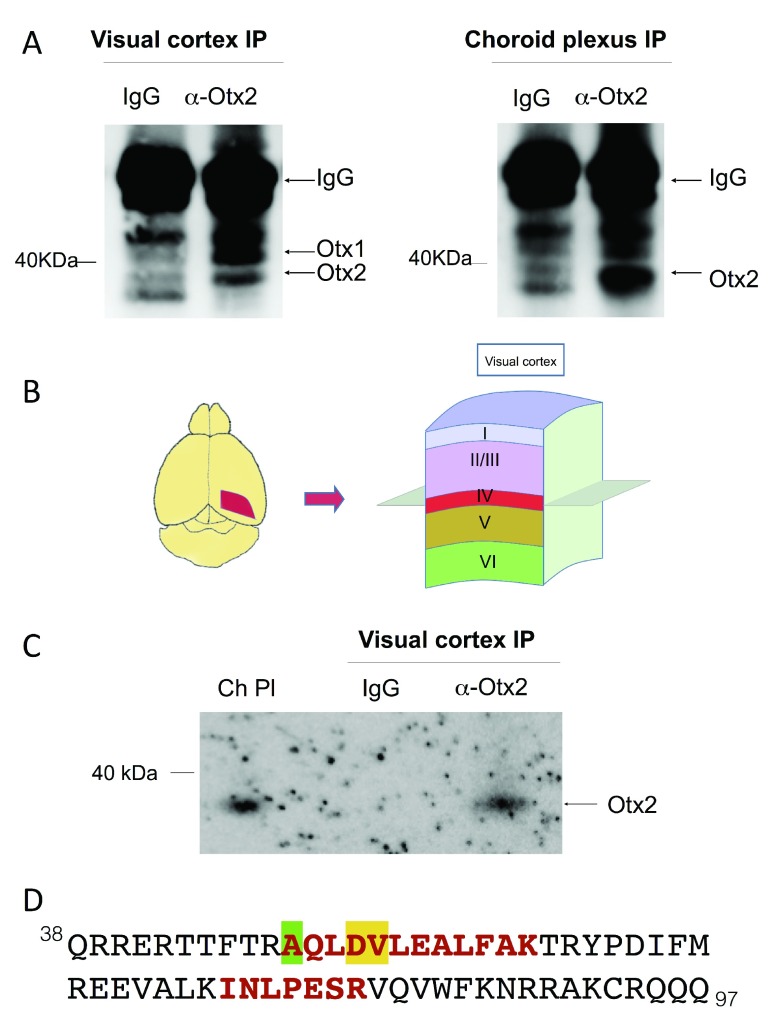
Otx2 protein in the granular and supragranular layers of adult mouse visual cortex. ( **A**) Immunoblots for Otx1/2 of immunoprecipitation (IP) experiments on extracts from visual cortex and choroid plexus. ( **B**) Diagram of finely dissected region for extracts containing granular (IV) and supragranular (I-III) layers of visual cortex. ( **C**) Immunoblot for Otx1/2 of samples from IP using cross-linked magnetic beads and finely dissected extracts. Choroid plexus (Ch Pl) extract was used to control for Otx2 migration velocity. ( **D**) The peptides (red, bold) matching Otx2 protein identified by high-resolution mass spectrometry. Only the homeodomain sequence of Otx2 (amino acids 38–97) is shown. The amino acid differing in Otx1 is highlighted in green, while amino acids that differ in Crx are highlighted in green and in yellow.

In order to analyze Otx homeoprotein distribution in the visual cortex, we turned to immunoprecipitation (IP) experiments. We first performed IP on whole visual cortex extracts, which showed both Otx1 and Otx2 protein ( Figure 2A). This result is confirmed by IP of choroid plexus, which strongly expresses Otx2 but only very weakly expresses Otx1. To analyze granular and supragranular content, we dissected and extracted the superior layers of posterior adult mouse cortex ( Figure 2B) and performed IP by using cross-linked magnetic beads. Immunoblot analysis detected only Otx2 but not Otx1 protein ( Figure 2C). This result was confirmed by mass spectrometry analysis on these extracts, which identified 2 Otx2 peptides (6.6% coverage with 100% specificity, Figure 2D). While this number of peptides is low, it was expected given that Otx2 is predicted to be poorly ionizable. Furthermore, identification has an error of 1 protein in 20,000 (19,999 are true) with a FDR of less than 0.5% for 2 peptides in our database of 16,671 sequences, thus the chance of misinterpretation is very low. These peptides are 100% specific for Otx2 and they have the same ions as the reference spectrum from a sample of purified Otx2 protein. While the second peptide is also specific for Otx1 and Crx homeoproteins, the first peptide is unique for Otx2 ( Figure 2D). These results confirm that the homeoprotein localized in granular and supragranular FSPV cells is indeed Otx2 and not Otx1.

## Discussion

The non-cell autonomous activity of homeoprotein transcription factors is now well established. There are clear phenotypes with recently developed *in vivo* single-chain secreted antibodies that neutralize extracellular homeoproteins yet leave intact cell autonomous activities ^9– 11^. Non-autonomy can also be demonstrated by comparing mRNA and protein expression. Indeed, the absence of mRNA in presence of the protein argues in favor of non-cell autonomy. However, when the receiving territory is a short distance from the producing territory, one could invoke the possibility of cell migration or mRNA instability to bring into question the reality of homeoprotein transfer.

In the visual system, Otx2 protein is found in the visual cortex far from two potential sources of Otx2 (where the *Otx2* locus is active), namely the eye and the choroid plexus ^5, 12^. Indeed, the *Otx2* locus is not active in the adult cerebral cortex as verified by using the *Otx2 ^+/GFP^* mouse, quantitative RT-PCR and *in situ* hybridization ^4^. In addition, conditional *Otx2* ablation in the choroid plexus reduces its content in FSPV cells, further supporting non-cell autonomy ^5^.

However, since most Otx1 and Otx2 antibodies are pan-Otx antibodies, it was still conceivable that some of the protein seen in FSPV cells by immunohistochemistry could correspond to Otx1 expressed in layer V of the cerebral cortex and transferred into PV cells. The present study shows that the staining in layer IV is maintained in the *Otx1* knockout mouse and that IP experiments of layers I-IV give immunoblot bands with expected Otx2 size and can be used to identified Otx2 by mass spectrometry. These results confirm that FSPV cells in granular and supragranular layers of the cerebral cortex only contain non-cell autonomous Otx2 and do not contain Otx1.

It may seem surprising that Otx1 expressed in layer V is not secreted and internalized by FSPV cells. Indeed the protein presents a homeodomain nearly identical to that of Otx2 and thus contains the two sequences necessary for internalization and secretion (for review see ^7^). However, previous studies have demonstrated that homeoproteins are transported from the basolateral to the apical side of polarized cells and thus into the axon ^7, 13^. Given their polarity and orientation, the pyramidal cells of layer V that express Otx1 are thus very unlikely to release it at the level of FSPV cells. In contrast, the choroid plexus epithelial cells present their apical surface toward the ventricles allowing Otx2 secretion into the cerebral spinal fluid. In conclusion, this study demonstrates that Otx2 is the only non-cell autonomous Otx family protein in the granular and supragranular FSPV cells.

## Data availability

*F1000Research*: Dataset 1. Quantitative PCR and mass spectrometry data of Otx2 homeoprotein in the mouse visual cortex, 10.5256/f1000research.4869.d33384 ^16^
