# Supplementary material for: Immunoprecipitation and mass spectrometry identify non-cell autonomous Otx2 homeoprotein in the granular and supragranular layers of mouse visual cortex
Source: F1000Res. 2014 Jul 30;3:178. [Version 1] doi: 10.12688/f1000research.4869.1 (PMC4133762; doi:10.12688/f1000research.4869.1)
Supplement: Peer review history

# Peer review: Referee response for version 1

**DOI:** 10.5256/f1000research.5197.r5636

**Published:** 2014-08-08

**Authors:** Paola Bovolenta

**Affiliations:** 1 Centro de Biología Molecular Severo Ochoa, CSIC-UAM, Universidad Autonoma de Madrid, Madrid, Spain

Studies, mostly from A. Prochiantz’s laboratory, have demonstrated that the transcription factor (TF) Otx2, produced by external sources such as the choroid plexus, is internalized into parvalbumin neurons of layers II/III and IV of the visual cortex, thereby controlling its plasticity. So far these studies had not ruled out the possibility that part of this effect could be mediated by Otx1, a closely related TF, which may act redundantly with Otx2. In this study, the authors combined the use of *Otx1* ^-/-^ mice with immunoprecipitation from layers I-IV extracts and high-resolution mass spectrometry analysis to demonstrate the sole presence of Otx2 peptides in these layers. The study thus demonstrates that Otx2 is the only non-cell autonomous Otx family member functional in fast-spiking parvalbumin cells of layers I-IV of the visual cortex.

This is a clear and well-executed study that solves an existing concern.

I have read this submission. I believe that I have an appropriate level of expertise to confirm that it is of an acceptable scientific standard.

# Peer review: Referee response for version 1

**DOI:** 10.5256/f1000research.5197.r5640

**Published:** 2014-08-04

**Authors:** Fred Gage

**Affiliations:** 1 Laboratory of Genetics, Salk Institute for Biological Studies, La Jolla, CA, USA

The authors address an issue that is unresolved in the literature. Is the Otx found in the cortex, and responsible in part of cortical plasticity, cortical derived Otx1 or choroid plexus derived Otx2? The problem exists because the antibodies used previously can not distinguish between the two. In the present study using *Otx1 *KO mice and a variety of measures, the authors prove Otx2 is the protein in the cortical cells and that this therefore is a cell non- autonomous phenomenon. The one question I have relates to figure 1B, which suggests there is greater Otx2 staining in the *Otx1 *KO mouse cortex. If this is a reliable observation the authors should comment. The low peptide coverage in the MASS SPEC could be a concern but the authors address that and in addition the other supporting data decrease the concern.

This is a clear, straightforward manuscript that adds important and definitive information to the existing literature

I have read this submission. I believe that I have an appropriate level of expertise to confirm that it is of an acceptable scientific standard.
